# The Effects of Poria cocos on Rho Signaling-Induced Regulation of Mobility and F-Actin Aggregation in MK-801-Treated B35 and C6 Cells

**DOI:** 10.1155/2022/8225499

**Published:** 2022-07-12

**Authors:** Yi-Chyan Chen, Chang-Ti Lee, Fu-Ming Tsai, Mao-Liang Chen

**Affiliations:** ^1^Department of Psychiatry, Taipei Tzu Chi Hospital, Buddhist Tzu Chi Medical Foundation, New Taipei City, Taiwan; ^2^Department of Psychiatry, School of Medicine, Tzu Chi University, Hualien, Taiwan; ^3^Department of Chinese Medicine, Taipei Tzu Chi Hospital, Buddhist Tzu Chi Medical Foundation, New Taipei City, Taiwan; ^4^Department of Research, Taipei Tzu Chi Hospital, Buddhist Tzu Chi Medical Foundation, New Taipei City, Taiwan; ^5^Department of Microbiology, Soochow University, Shih Lin, Taipei City, Taiwan

## Abstract

**Methods:**

B35 neuronal cells and C6 glial cells were incubated with MK-801 for 7 days followed by MK-801, MK801 in combination with water extracts of P. cocos (PRP for P. cocos cum Radix Pini or WP for White Poria) treatment for an additional 7 days. Analysis of cell mobility, F-actin aggregation, and Rho signaling modulation was performed to clarify the roles of PRP or WP in MK-801-treated B35 and C6 cells.

**Results:**

MK-801 decreases B35 cell mobility, whereas the inhibited cell migration ability and F-actin aggregation in MK-801-treated B35 or C6 cells could be reversed by PRP or WP. The CDC42 expression in B35 or C6 cells would be reduced by MK-801 and restored by treating with PRP or WP. The RhoA expression was increased by MK-801 in both B35 and C6 cells but was differentially regulated by PRP or WP. In B35 cells, downregulation of PFN1, N-WASP, PAK1, and ARP2/3 induced by MK-801 can be reversely modulated by PRP or WP. PRP or WP reduced the increase in the p-MLC2 expression in B35 cells treated with MK-801. The reduction in ROCK1, PFN1, p-MLC2, and ARP2/3 expression in C6 cells induced by MK-801 was restored by PRP or WP. Reduced N-WASP and PAK1 expression was differentially regulated by PRP or WP in MK-801-treated C6 cells.

## 1. Introduction

Cytoskeletal reorganization can lead to changes in cell function [1], including elongation of neuronal spines of synapse, cell mobility [2–4], and plasticity of neuron [5, 6]. These cell functions play critical roles in developing neurons and involve the identical regulatory signaling pathway named the Rho signaling pathway. The Rho signaling pathway is triggered by the activation of Rho family proteins and regulates biological functions, such as gene transcription regulation, membrane trafficking, growth/shrinkage of microtubules, and actin cytoskeleton reorganization. The most studied proteins in all Rho family protein members are Ras homolog family member A (RhoA), Ras-related C3 botulinum toxin substrate 1 (Rac1), and cell division cycle 42 (Cdc42), which mediate the formation of stress fibers, lamellipodia formation, membrane ruffling, the formation of filopodia/microspikes, and neuronal development [7]. A previous study suggested that the abnormality of recognitive function in animals might be caused by the impairment of hippocampal neurons [8]. Schubert et al. [9] mentioned that activated RhoA-GTPase induces the regulation of dendritic spine morphology in cultured hippocampal neurons. A reduction in CDC42 expression levels is accompanied by reduced dendritic spine density in the brains of schizophrenic patients. A recent study also proposed that ketamine-induced reductions in Rho signaling might be related to impairment of cognition in schizophrenic patients [10].

Addictive behaviors have been proposed to be closely related to cognitive function [11, 12]. Drug addiction has also been reported to be an affective-cognitive disorder with dopamine transmission abnormalities [13] and N-methyl-d-aspartate receptor (NMDAR) activity [14]. The neuropathology of drug addiction has been suggested to involve cognitive functions, such as memory, learning, attention, and inhibitory control during the development of drug dependence [15]. Previous studies revealed that ketamine would reduce RhoA and ROCK1 expression levels to further cause a reduction of mushroom spine formation and stubby spine number of hippocampal neurons in rats, which might be related to impaired cognitive function in schizophrenic patients [8, 10, 16–18]. It has also been reported that RhoA might interact with the metabotropic glutamate receptor 1, *α*-amino-3-hydroxy-5-methyl-4-isoxazolepropionic acid receptor, and NMDAR to maintain the stabilization of NMDAR and to modulate the reconstruction of spine actin at excitatory postsynapses [9]. In addition, the regulation of dendritic spine morphology in cultured hippocampal neurons can be induced by activating RhoA [9]. These findings suggested that addictive behaviors might be related to cognitive functions that can be modulated by the Rho signaling pathway.

Poria *cocos* is a traditional Chinese medicine containing two major ingredients, triterpenoids and polysaccharides, and some minor chemical substances, including potassium salts, amino acids, choline, histidine, and steroids [19, 20]. *P. cocos* cum Radix Pini (PRP; Sclerotium paradicis, named *Fu Shen*) and White Poria (WP; named *Bai Fu Ling*) are two commonly used herbal medicines of *P. cocos* with pharmacological anti-inflammatory properties and immunomodulatory properties [21–23]. Several reports mentioned that the extract of *P. cocos* regulates the cytoplasmic free calcium concentration in the brain neurons of neonatal rats and dose-dependently modulates glutamate-induced cytosolic free calcium [19, 23]. Some studies have also suggested that cytoplasmic free calcium in cells can regulate the Rho signaling pathway to further modulate cell functions, such as directed movement, mesoderm migration, cytoskeletal reorganization, neuronal cell plasticity, and cancer metastasis [2, 6, 24–26]. The impairment of specific synaptic plasticity in the mesolimbic dopamine system, which is central to reward processing in the brain, was suggested to be related to drug abuse behaviors [27]. Our previous study revealed that the water extract of *Poria cocos* can modulate cytoskeletal reorganization and cell migration by affecting RhoA and CDC42 and the subsequent Rho signaling pathway. The water extract of *Poria cocos* could also reverse ketamine-induced effects on cytoskeleton reorganization and cell migration by regulating the RhoA, CDC42, and Rho signaling pathways [28].

MK-801 is an NMDA antagonist that can desensitize addictive behaviors to ethanol, morphine, and cocaine [29, 30]. MK-801 can also induce negative symptoms of schizophrenia, such as cognitive disruption, reduction of long-term potentiation, and learning defects [31–33]. Various previous studies have mentioned that glial cells can maintain neuronal cell function by providing cell shape maintenances, nutrients, growth factors, and recycling of neurotransmitters. The differential effects of *Poria cocos* on these two different types of cells should also be clarified to further understand the drug effects exhibited by *P. cocos* on different cells in the brain. In this study, we aimed to investigate the regulatory effects of *P. cocos* (PRP and WP) on Rho signaling pathway regulation in MK-801-treated B35 neuronal cells and C6 glial cells. We revealed that Rho family proteins (RhoA and CDC42) and Rho signaling-related proteins in B35 and C6 cells could be affected by treating with PRP or WP. We also found either of PRP or WP could recover the inhibitory effects on cell mobility and actin aggregation induced by MK-801 in B35 or C6 cells. Our data proposed that PRP or WP-induced regulation of cell mobility and F-actin reorganization in B35 and C6 cells treated with MK-801 might be caused by reversely modulating RhoA, CDC42, and further Rho signaling pathway regulation in cells.

## 2. Materials and Methods

### 2.1. PRP and WP Water Extract Preparation

PRP and WP herbal powder extracts were purchased from Sun Ten Pharmaceutical Company (New Taipei City, Taiwan). For the preparation of the PRP and WP solutions, the PRP or WP herbal powders were weighed and extracted using sterilized ddH2O for 6 h at room temperature. The stock concentration of each of the PRP and WP was adjusted to10 mg/ml. After centrifugation, collect the corresponding supernatant from the PRP and WP solution and store −20°C for future use.

### 2.2. B35 and C6 Cell Culture and Drug Treatments

MK-801 was purchased from Sigma–Aldrich (St. Louis, USA). B35 and C6 cells were obtained from the Bioresource Collection and Research Center (BCRC) of the Food Industry Research and Development Institute (FRDI), Taiwan. For B35 cells, cells were cultured in MEM (Invitrogen, Life Technologies) containing 10% fetal bovine serum (Invitrogen, Life Technologies), 2 mM L-glutamine, 100 *μ*g/ml streptomycin, and 100 U/ml penicillin. High-glucose Dulbecco's Modified Eagle's Medium (Invitrogen, Life Technologies Incorporation, Eugene, OR, USA) supplemented with 2 mM L-glutamine, 2% fetal bovine serum (Invitrogen Life Technologies) and 10% heat-inactivated horse serum (Invitrogen Life Technologies) were used to maintain C6 cells throughout the experiments. To examine the effects of MK-801, PRP, and WP on B35 and C6 cells, cells were treated with MK-801 daily for 7 days followed by the addition of MK-801 alone or in combination with either PRP or WP for an additional 7 days. The final drug concentration used was 25 *μ*M for MK-801, 10 *μ*g/ml for PRP, and 10 *μ*g/ml for WP. B35 or C6 cells were then collected for extracting total protein, followed by subsequent expression level analysis of RhoGDI1, Rho family proteins (RhoA and CDC42), and Rho signaling pathway-related proteins (ROCK1, PFN1, p-MLC2, N-WASP, ARP2/3, and PAK1).

### 2.3. Western Blot Analysis

Total cell lysates were prepared by lysing B35 or C6 cells in mammalian protein extraction buffer (GE Healthcare Bio-Science, Uppsala, Sweden) supplemented with Protease Inhibitor Mix (GE Healthcare Bio-Science). To examine the protein expression levels, 5–80 *μ*g of total protein extract of B35 or C6 was analyzed by separating in 10-15% sodium dodecyl sulfate polyacrylamide gels accordingly. The polyvinylidene difluoride membranes were used for transferring separated proteins. The membrane was then blocked with Membrane Blocking Solution (Life Technology, Frederick, MD, USA) for 1 h. The blocked membranes were then incubated with specific primary antibodies at 4°C for 12 h, followed by incubation with the respective horseradish peroxidase-conjugated secondary antibodies at room temperature for 4 h. Amersham ECL kit (Amersham, Bucks, UK) was used to develop and to reveal the signals of protein bands.

### 2.4. Mobility Analysis of B35 and C6 Cells

Before performing the cell mobility assay, B35 or C6 cells were cultured in medium with MK-801 for 7 days, followed by treating with MK-801 combined with PRP or WP for another 5 days. Drug-treated B35 cells (10^4^ cells/well) or drug-treated C6 (5 × 10^3^ cells/well) were added to the upper Transwell (Costar, Coring Incorporation, Kennebunk, ME, USA) insert compartment with an 8 *μ*m pore size polycarbonate membrane. Cells in Transwell insert were then incubated in a 24-well tissue culture plate with medium containing MK-801 and either PRP or WP for another 2 days. Cell migration assays were performed by fixing B35 or C6 cells with methanol and followed by staining the cells with a propidium iodide solution (Sigma) (50 *μ*g/ml) and staying at room temperature for 45 min. The migrated cells on the other side of the membrane were counted using a fluorescent microscope at ×40 magnification. The mobility assay for all experiments in this study was performed independently in triplicate.

### 2.5. Analysis of Actin Condensation in B35 and C6 Cells

B35 or C6 cells were cultured with MK-801 for 7 days and then incubated with MK-801 in combination with or without PRP/WP for another 5 days. 5 × 10^3^ drug-treated B35 or C6 cells were then seeded in 6-well plate coverslips coated with poly-L-lysine. The cells were further cultured for another 2 days in medium with corresponding MK-801, PRP, or WP. The coverslips with drug-treated B35 or C6 cells were moved to a new 6-well plate and fixed by incubating the coverslips in methanol for 2 h at -20°C. After washing with PBS, the cells on coverslips were stained by immersing in ActinGreen™ 488 RreadyProbes™ reagent (Invitrogen, Life Technologies) at room temperature for 90 min according to the manufacturer's suggestion. The traced dye was washed out from the cells by using PBS, and the coverslips were moved to glass slides and mounted with SlowFade™ Diamond Antifade Mountant (Invitrogen, Life Technologies). The images were then captured on a fluorescent microscope at ×40 magnification.

### 2.6. Quantification of Protein Expression Level and Statistical Analysis

The expression level of the examined proteins in all western blot experiments was obtained by detecting the density of developed bands using ImageJ software (https://imagej.nih.gov/ij/). Differences in normalized protein expression levels and cell migration assays between MK-801 and control, differential drug-treated B35 and C6 cells were analyzed by using Student's *t*-test. A *p* value less than 0.01 (^∗∗^) or 0.05 (^∗^) was used to represent significant differences between compared groups.

## 3. Results

### 3.1. PRP and WP Modulate MK-801-Induced RhoGDI1, RhoA, and CDC42 Regulation

**Figure 1:**
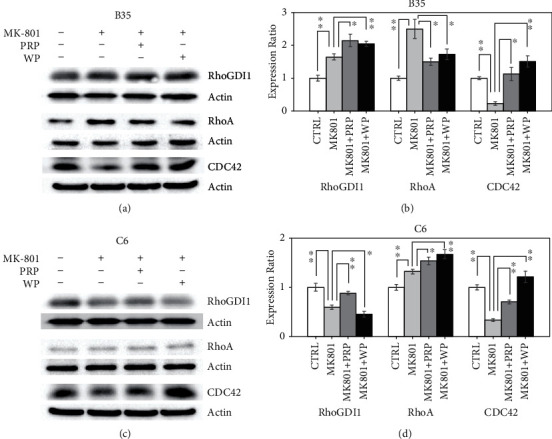
PRP and WP differentially regulate MK-801-induced RhoGDI1, RhoA, and CDC42 regulation. Western blotting revealed the expression changes of RhoGDI1, RhoA, and CDC42 induced by PRP/WP in (a) B35 and (c) C6 cells treated with MK-801. The protein expression was quantified by using ImageJ software. Beta-actin was used as a normalization control to calculate the relative expression of the examined targets. The bar chart was constructed according to the data of three independent western blot experiments that analyzed three different batches of protein extracts of drug-treated (b) B35 and (d) C6 cells. The data was analyzed by using Student's *t*-test (^∗^*p* value<0.05; ^∗∗^*p* value<0.01) analysis.

Our previous findings revealed that PRP and WP could modulate the expression levels of RhoGDI1 and RhoA and CDC42 but not Rac1 proteins [28]. In the present study, we examined the effects of PRP and WP on modulating the RhoGDI1, RhoA, and CDC42 protein expression in MK-801-treated B35 and C6 cells. We found that MK-801 increased the RhoGDI1 expression (*p* value <0.01) and was further increased by PRP (*p* value<0.05) and WP (*p* value <0.01) in B35 cells (Figures 1(a) and 1(b)). The increased RhoA expression induced by MK-801 (*p* value <0.01) in B35 cells was restored by PRP (*p* value <0.05) and WP (*p* value <0.05) (Figures 1(a) and 1(b)). In contrast, we found that the MK-801-induced reduction in the RhoGDI1 expression (*p* value <0.01) was recovered by PRP (*p* value <0.01) but was further reduced by WP (*p* value <0.05) in C6 cells (Figures 1(c) and 1(d)). The RhoA expression induced by MK-801 treatment in C6 cells (*p* value <0.01) was further increased by PRP (*p* value <0.05) and WP (*p* value <0.01) (Figures 1(c) and 1(d)). The reduction in the CDC42 expression in MK-801-treated B35 (*p* value <0.01) and C6 (*p* value <0.01) cells was recovered by either PRP (*p* value <0.05 for B35 and *p* value <0.01 for C6) or WP (*p* value <0.01 for both cells) treatment (Figure 1).

### 3.2. Effects of PRP and WP on Modulating F-Actin Reorganization in MK-801-Treated B35 and C6 Cells

**Figure 2:**
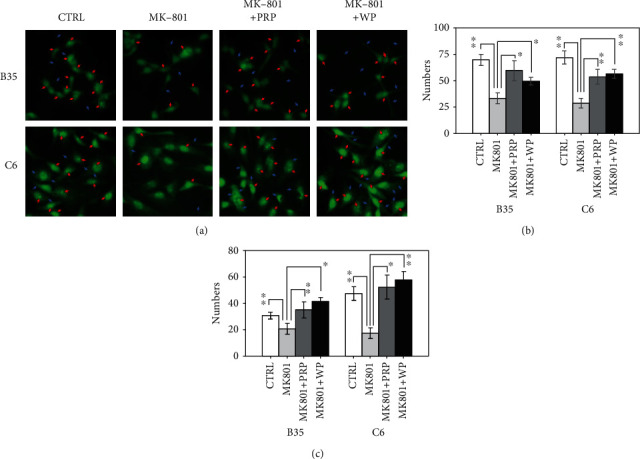
Effects of PRP and WP on actin filament reorganization in B35 and C6 cells treated with MK-801. MK-801-treated B35 and C6 cells on coverslips were incubated with PRP or WP accordingly. ActinGreen™ 488 RreadyProbes™ reagent was used for F-actin in staining in B35 and C6 cells. (a) Red arrows show actin nucleation and blue arrows show F-actin condensation. The numbers of (b) actin nucleation and (c) F-actin condensation were obtained by counting the condensed actin spot in nuclei or condensed actin filament in extended-cytoplasm in 100 cells under different fields of view. The bar charts were made from three independent batches of experiments and analyzed by using Student's  *t*-test (^∗^*p* value <0.05; ^∗∗^*p* value <0.01) analysis.

Actin condensation and cytoskeletal reorganization play important roles in various cell functions mediated by Rho signaling, including actin nucleation/polymerization, regulation of cell shape, microtubule formation, and cell polarity regulation. We observed that MK-801 could reduce actin nucleation in both B35 and C6 cells after staining cells with phalloidin (Figure 2). We also observed a reduction in actin filament formation in B35 and C6 cells upon treatment with MK-801 (Figure 2). Both PRP and WP reversed the inhibitory effects of MK-801 on actin nucleation and F-actin construction in B35 and C6 cells.

### 3.3. PRP and WP Induced RhoA-Related Rho Signaling Regulation in MK-801-Treated B35 and C6 Cells

**Figure 3:**
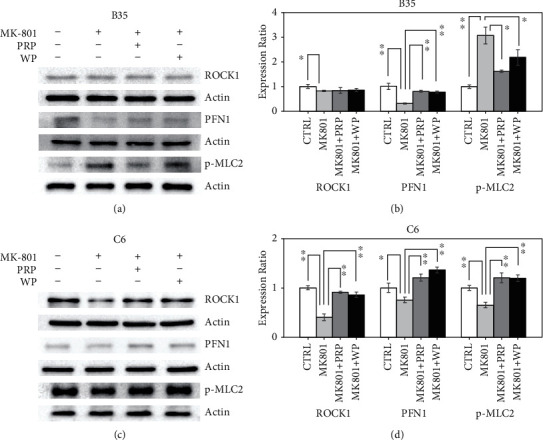
PRP and WP regulate MK-801-induced ROCK1, PFN1, and p-MLC2 regulation. Western blotting revealed the expression changes of ROCK1, PFN1, and p-MLC2 induced by PRP/WP in (a) B35 and (c) C6 cells treated with MK-801. The protein expression was quantified by using ImageJ software. Beta-actin was used as a normalization control to calculate relative expression of examined target. The bar chart was constructed according to the data of three independent western blot experiments that analyzed three different batches of protein extracts of drug-treated (b) B35 and (d) C6 cells. The data was analyzed by using Student's *t*-test (^∗^*p* value <0.05; ^∗∗^*p* value <0.01) analysis.

In RhoA-regulated Rho signaling pathway, ROCK1, profilin 1 (PFN1), and phosphorylated myosin light chain 2 (p-MLC2) are proteins that can modulate F-actin assembly and condensation. The ROCK1 expression was decreased by MK-801 (*p* value<0.05) in B35 cells (Figures 3(a) and 3(b)), whereas PRP and WP did not affect the reduced ROCK1 level caused by MK801. The reduction in the ROCK1 expression caused by MK-801 (*p* value<0.01) in C6 cells could be increased by either PRP (*p* value <0.01) or WP (*p* value <0.01) (Figures 3(c) and 3(d)). The reduction in the PNF1 expression induced by MK-801 in B35 (*p* value <0.01) (Figures 3(a) and 3(b)) and C6 (*p* value <0.05) (Figures 3(c) and 3(d)) cells could be recovered by PRP (*p* value <0.01 for both B35 and C6 cells) and WP (*p* value <0.01 for both B35 and C6 cells). The increased expression of p-MLC2 in MK-801-treated B35 cells (*p* value <0.01) was reduced by PRP (*p* value <0.05) and WP (*p* value <0.05) (Figures 3(a) and 3(b)). In contrast, we observed that MK-801 reduced the p-MLC2 expression (*p* value <0.01) in C6 cells, which could be recovered by PRP (*p* value <0.01) and WP (*p* value <0.01) (Figures 3(c) and 3(d)).

### 3.4. PRP and WP Restored MK-801-Mediated Inhibition of B35 and C6 Cell Migration

**Figure 4:**
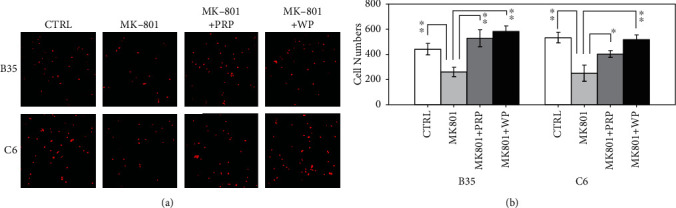
Effects of MK-801, PRP, and WP on B35 and C6 cell migration. (a) Cell mobility of B35 and C6 cells was analyzed. The migrated cells were stained with propidium iodide, and then the number of cells on the membrane was counted. (b) The result in the bar chart was made from cell counts of three different drug-treated cell batches and analyzed using Student's *t*-test (^∗^*p* value <0.05; ^∗∗^*p* value <0.01).

To examine the effect of PRP and WP on MK-801-induced inhibition of B35 and C6 cell migration, B35 or C6 cells were incubated with MK-801 for 7 days followed by PRP or WP treatment for another 6 days. Then, a cell migration assay was performed for an additional 24 h. As shown in Figure 4, B35 and C6 cell migration was inhibited by MK-801 (*p* value <0.01 for both B35 and C6 cells). However, MK-801-induced inhibition of B35 (*p* value <0.01 for both PRP and WP) and C6 cell migration (*p* value <0.01 for both PRP and WP) could be restored by PRP and WP.

### 3.5. PRP and WP Modulated CDC42-Related Rho Signaling Regulation in MK-801-Treated B35 and C6 Cells

**Figure 5:**
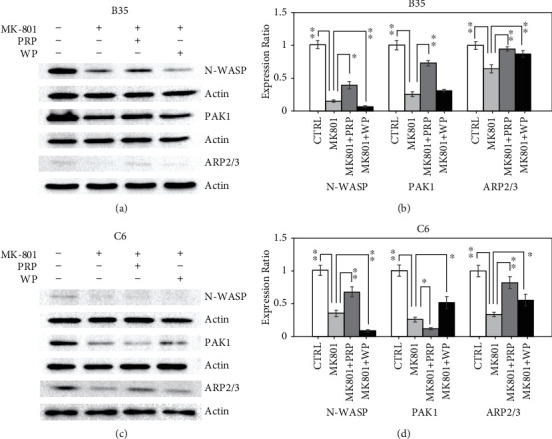
PRP and WP regulate MK-801-induced N-WASP, PAK1, and ARP2/3 regulation. Western blotting revealed the expression changes of the indicated proteins induced by PRP/WP in (a) B35 and (c) C6 cells treated with MK-801. The protein expression was quantified by using ImageJ software. Beta-actin was used as a normalization control to calculate relative expression of examined target. The bar chart was constructed according to the data of three independent western blot experiments that analyzed three different batches of protein extracts of drug-treated (b) B35 and (d) C6 cells. The data was analyzed by using Student's *t*-test (^∗^*p* value <0.05; ^∗∗^*p* value <0.01) analysis.

Activation of CDC42 can modulate cell migration ability by regulating neuronal Wiskott–Aldrich syndrome protein (N-WASP), p21 (RAC1)-activated kinase 1 (PAK1), and RhoA protein–modulated actin-related protein 2/3 (ARP2/3) to further regulate various cell functions, including actin polymerization, filopodia, and cell migration. We observed that the reduction in the N-WASP expression in MK-801-treated B35 (Figures 5(a) and 5(b)) and C6 cells (Figures 5(c) and 5(d)) (*p* value <0.01 for both B35 and C6 cells) was restored by PRP (*p* value<0.05 for B35 cells and *pv*alue <0.01 for C6 cells) but was further reduced by WP (*p* value <0.01 for both B35 and C6 cells). PRP (*p* value <0.01) but not WP enhanced the reduction in the PAK1 expression in B35 cells treated with MK-801 (*p* value <0.01) (Figures 5(a) and 5(b)). In MK-801-treated C6 cells, the downregulation of PAK1 (*p* value <0.05) was further reduced by PRP (*p* value <0.05) but will be enhanced by WP (*p* value <0.05) (Figures 5(c) and 5(d)). MK8-01 reduced the ARP2/3 expression in B35 (*p* value <0.01) (Figures 5(a) and 5(b)) and C6 cells (*p* value <0.01) (Figures 5(c) and 5(d)), whereas both PRP (*p* value<0.01 for both B35 and C6 cells) and WP (*p* value <0.01 for B35 cells and *p* value <0.05 for C6 cells) restored ARP2/3 expression levels.

## 4. Discussion

The Rho signaling pathway plays important roles in modulating actin filament construction to regulate various cell functions, such as cell shape changes, cell migration, neuronal cell plasticity, cytoskeleton reorganization, and microtubule formation. The relationship between Rho signaling regulation and neuronal cell plasticity has been mentioned and well studied in various studies. Many studies have also revealed that neuronal cell plasticity is related to the generation of addictive behaviors [27, 34]. MK-801 was found to impair cognitive function, learning ability, and memory and was also used to ease addictive behaviors [29–33, 35]. The present study revealed that MK-801 could enhance the RhoA expression and reduce the CDC42 expression in both B35 and C6 cells. We also found that PRP and WP could reverse the effects of the MK-801 on CDC42 expression in both cell types. This finding suggested that PRP and WP might regulate CDC42 but not RhoA through a similar mechanism in MK-801-treated B35 and C6 cells.

Although MK-801 was found to ease the addictive behaviors induced by various drugs, MK-801 was also found to impair recognition function by regulating neuronal plasticity and related immediate early gene expression by inhibiting NMDA receptors on pyramidal neurons and axonal boutons in hippocampal interneurons of rats [36–38]. Recently, MK-801 was revealed to decrease AMPA receptors and further metaplasity (plasticity of synaptic plasticity) of neurons [39, 40] related to stress or drugs of abuse. In this study, PRP and WP were found to reverse regulation of RhoA and CDC42 expression level in MK-801-treated B35 neuronal cells. Furthermore, PRP and WP were also found to reverse MK-801-induced PFN1, pMLC2, and ARP2/3 expression in B35 cells. Regulation of N-WASP and PAK1 in MK-801-treated B35 cells could be restored by PRP but not WP. The differential regulation of N-WASP and PAK1 may be caused by the different ingredients of PRP and WP. The regulatory effects of the ingredients PRP and WP on Rho signaling proteins should be further studied.

Activation of ROCK1 protein by activated RhoA might further induce the phosphorylation of p-MLC2. A recent study observed that directly delivering Fasudil, a ROCK protein inhibitor, to the prefrontal cortex of mice might enhance goal-directed behavior and block the habitual response to cocaine [41]. Our study has shown that the MK-801-induced ROCK1 expression was not affected by PRP or WP in B35 cells. Interestingly, we also observed the decreased PFN1 and increased p-MLC2 expression in MK-801-treated B35 cells, which could be reversed by PRP and WP. The regulatory trends were the same as those of RhoA expression regulation. This finding suggested that the ROCK1 expression might be regulated by factors other than RhoA activation.

MK-801 is a NMDAR antagonist that can bind NMDAR and to further induce changes of calcium level in cytoplasm of cells. Although both B35 neuronal cell and C6 glial cell have NMDARs for binding of MK-801, the proportion of NMDARs on the membrane and the modulation of signaling downstream the NMDARs might be varied between different cell types to induce differential RhoGDI1 regulation. These might be the reasons that RhoGDI1 expressions were differentially regulated in B35 cell and C6 cell. In addition, the different effects of PRP and WP on the regulation of the RhoA expression in B35 and C6 cells may be caused by the different susceptibility of the cells to PRP and WP. Furthermore, the ingredients of PRP and WP are similar but still slightly different. This might be the factor that PRP and WP induce differential regulation of RhoGDI1, N-WASP, and PAK1 expression in C6 cells. To determine whether the differences of ingredients between PRP and WP might be the reason to induce differential regulation of RhoGDI1, N-WASP, and PAK1 in B35 and/or in C6 cells should be further studied. Additionally, RhoA, CDC42, and Rac1 separately and dynamically modulate actin filament formation, actin contraction, and lamellipodial protrusions in cell. LIMK was found that can be coregulated by RhoA- and CDC42-related Rho signaling. The crosstalk between RhoA- or CDC42-related signaling via LIMK might be the cause for the inconsistent regulation between ROCK1, PFN1/p-MLC2, N-WASP, and PAK1.

## 5. Conclusion

The relationships between addictive behaviors and Rho signaling (except RhoA, CDC42, and ROCK1) remain unclear. We conclude that PRP and WP could regulate the RhoA and CDC42 expression to modulate Rho signaling pathway and follow cell migration, actin nucleation, and F-actin remodeling in MK-801-treated B35 and C6 cells. Further studies should be also performed to better clarify the roles of PRP/WP playing on Rho signaling, neuronal plasticity, and addictive behaviors in suitable animal models.

## Data Availability

All data used to support the findings of this study are available from the corresponding author upon reasonable request.

## References

[1] Fletcher D. A., Mullins R. D. (2010). Cell mechanics and the cytoskeleton. *Nature*.

[2] Tsai F. C., Kuo G. H., Chang S. W., Tsai P. J. (2015). Ca2+ signaling in cytoskeletal reorganization, cell migration, and cancer metastasis. *BioMed Research International*.

[3] Tang D. D., Gerlach B. D. (2017). The roles and regulation of the actin cytoskeleton, intermediate filaments and microtubules in smooth muscle cell migration. *Respiratory Research*.

[4] Yamazaki D., Kurisu S., Takenawa T. (2005). Regulation of cancer cell motility through actin reorganization. *Cancer Science*.

[5] Nakahata Y., Yasuda R. (2018). Plasticity of spine structure: local signaling, translation and cytoskeletal reorganization. *Frontiers in Synaptic Neuroscience*.

[6] Kuriu T., Inoue A., Bito H., Sobue K., Okabe S. (2006). Differential control of postsynaptic density scaffolds via actin-dependent and -independent mechanisms. *Journal of Neuroscience*.

[7] Hall A. (1998). Rho GTPases and the actin cytoskeleton. *Science*.

[8] Ivy A. S., Rex C. S., Chen Y. (2010). Hippocampal dysfunction and cognitive impairments provoked by chronic early-life stress involve excessive activation of CRH receptors. *Journal of Neuroscience*.

[9] Schubert V., Da Silva J. S., Dotti C. G. (2006). Localized recruitment and activation of RhoA underlies dendritic spine morphology in a glutamate receptor-dependent manner. *The Journal of Cell Biology*.

[10] Jiang S., Hao Z., Li X. (2018). Ketamine destabilizes growth of dendritic spines in developing hippocampal neurons in vitro via a Rhodependent mechanism. *Molecular Medicine Reports*.

[11] Gould T. J. (2010). Addiction and cognition. *Addiction Science & Clinical Practice*.

[12] D'Souza M. S. (2019). Brain and cognition for addiction medicine: from prevention to recovery neural substrates for treatment of psychostimulant-induced Cognitive deficits. *Frontiers in Psychiatry*.

[13] Fattore L., Diana M. (2016). Drug addiction: an affective-cognitive disorder in need of a cure. *Neuroscience & Biobehavioral Reviews*.

[14] Hopf F. W. (2017). Do specific NMDA receptor subunits act as gateways for addictive behaviors?. *Genes, Brain and Behavior*.

[15] Ersche K. D., Sahakian B. J. (2007). The neuropsychology of amphetamine and opiate dependence: implications for treatment. *Neuropsychology Review*.

[16] Buchtova H., Fajnerova I., Stuchlik A., Kubik S. (2017). Acute systemic MK-801 induced functional uncoupling between hippocampal areas CA3 and CA1 with distant effect in the retrosplenial cortex. *Hippocampus*.

[17] Han D., Xu L., Xiao H., Prado Schmidt G. C., Shi S. (2013). Dizocilpine reduces head diameter of dendritic spines in the hippocampus of adolescent rats. *Psychiatry Research*.

[18] Juliandi B., Tanemura K., Igarashi K. (2015). Reduced adult hippocampal neurogenesis and cognitive impairments following prenatal treatment of the antiepileptic drug valproic acid. *Stem Cell Reports*.

[19] Rios J. L. (2011). Chemical constituents and pharmacological properties of Poria cocos. *Planta Medica*.

[20] Feng Y. L., Zhao Y. Y., Ding F. (2013). Chemical constituents of surface layer of Poria cocos and their pharmacological properties (I). *Zhongguo Zhong Yao Za Zhi= Zhongguo Zhongyao Zazhi= China journal of Chinese Materia Medica*.

[21] Cuellar M. J., Giner R. M., Recio M. C., Just M. J., Manez S., Rios J. L. (1997). Effect of the basidiomycete Poria cocos on experimental dermatitis and other inflammatory conditions. *Chemical and Pharmaceutical Bulletin*.

[22] Spelman K., Burns J., Nichols D., Winters N., Ottersberg S., Tenborg M. (2006). Modulation of cytokine expression by traditional medicines: a review of herbal immunomodulators. *Alternative Medicine Review*.

[23] Yu S. J., Tseng J. (1996). Fu-Ling, a Chinese herbal drug, modulates cytokine secretion by human peripheral blood monocytes. *International Journal of Immunopharmacology*.

[24] Hayashi K., Yamamoto T. S., Ueno N. (2018). Intracellular calcium signal at the leading edge regulates mesodermal sheet migration during Xenopus gastrulation. *Scientific Reports*.

[25] Wei C., Wang X., Zheng M., Cheng H. (2012). Calcium gradients underlying cell migration. *Current Opinion in Cell Biology*.

[26] Sakurada S., Takuwa N., Sugimoto N. (2003). Ca2+-dependent activation of Rho and Rho kinase in membrane depolarization-induced and receptor stimulation-induced vascular smooth muscle contraction. *Circulation Research*.

[27] Kauer J. A., Malenka R. C. (2007). Synaptic plasticity and addiction. *Nature Reviews Neuroscience*.

[28] Lee C. Y., Lee C. T., Tzeng I. S., Kuo C. Y., Tsai F. M., Chen M. L. (2021). Poria cocos regulates cell migration and actin filament aggregation in B35 and C6 cells by modulating the RhoA, CDC42, and Rho signaling pathways. *Evidence-Based Complementary and Alternative Medicine*.

[29] Camarini R., Frussa-Filho R., Monteiro M. G., Calil H. M. (2000). MK-801 blocks the development of behavioral sensitization to the ethanol. *Alcoholism: Clinical and Experimental Research*.

[30] Jeziorski M., White F. J., Wolf M. E. (1994). MK-801 prevents the development of behavioral sensitization during repeated morphine administration. *Synapse*.

[31] Coan E. J., Saywood W., Collingridge G. L. (1987). MK-801 blocks NMDA receptor-mediated synaptic transmission and long term potentiation in rat hippocampal slices. *Neuroscience Letters*.

[32] Murray T. K., Ridley R. M., Snape M. F., Cross A. J. (1995). The effect of dizocilpine (MK-801) on spatial and visual discrimination tasks in the rat. *Behavioural Pharmacology*.

[33] Murray T. K., Ridley R. M. (1997). The effect of dizocilpine (MK-801) on conditional discrimination learning in the rat. *Behavioural Pharmacology*.

[34] Mameli M., Luscher C. (2011). Synaptic plasticity and addiction: learning mechanisms gone awry. *Neuropharmacology*.

[35] Brown T. E., Lee B. R., Sorg B. A. (2008). The NMDA antagonist MK-801 disrupts reconsolidation of a cocaine-associated memory for conditioned place preference but not for self-administration in rats. *Learning & Memory*.

[36] Czerniawski J., Ree F., Chia C., Ramamoorthi K., Kumata Y., Otto T. A. (2011). The importance of having Arc: expression of the immediate-early gene Arc is required for hippocampus-dependent fear conditioning and blocked by NMDA receptor antagonism. *Journal of Neuroscience*.

[37] Kubík Š., Buchtová H., Valeš K., Stuchlík A. (2014). MK-801 impairs cognitive coordination on a rotating arena (carousel) and contextual specificity of hippocampal immediate-early gene expression in a rat model of psychosis. *Frontiers in Behavioral Neuroscience*.

[38] Perez-Rando M., Castillo-Gómez E., Guirado R. (2017). NMDA receptors regulate the structural plasticity of spines and axonal boutons in jippocampal interneurons. *Frontiers in Cellular Neuroscience*.

[39] Piva A., Caffino L., Mottarlini F. (2021). Metaplastic effects of ketamine and MK-801 on glutamate receptors expression in rRat medial prefrontal cortex and hippocampus. *Molecular Neurobiology*.

[40] Zhao J., Peng Y., Xu Z. (2008). Synaptic metaplasticity through NMDA receptor lateral diffusion. *Journal of Neuroscience*.

[41] DePoy L. M., Zimmermann K. S., Marvar P. J., Gourley S. L. (2017). Induction and blockade of adolescent cocaine-induced habits. *Biological Psychiatry*.

